# Quantitative analysis of vertebral fat fraction and R2^*^ in osteoporosis using IDEAL-IQ sequence

**DOI:** 10.1186/s12891-023-06846-4

**Published:** 2023-09-11

**Authors:** Feng Zhou, Bo Sheng, Furong Lv

**Affiliations:** Department of Radiology, the First Affiliated Hospital of Chongqing Medical University, Yuzhong District, 1 Youyi Road, Chongqing, 400016 China

**Keywords:** IDEAL-IQ sequence, Fat fraction, R2*, Bone mineral density, Osteoporosis

## Abstract

**Objective:**

To investigate the correlation between FF, R2* value of IDEAL-IQ sequence and bone mineral density, and to explore their application value in the osteoporosis.

**Methods:**

We recruited 105 women and 69 men aged over 30 years who voluntarily underwent DXA and MRI examination of lumbar spine at the same day. Participants were divided into normal, osteopenia and osteoporosis group based on T-score and BMD value of DXA examination. One-way ANOVA was adopted to compare the quantitative parameters among the three groups. Independent samples t-test was utilized to compare FF and R2* value between men and women.Pearson correlation analysis was used to research the correlation between FF, R2* value and BMD.

**Results:**

Age, height, weight, BMD and FF value were significantly different among three groups (*p* < 0.05). No significant difference was found in FF value between male and female group, while R2* value were significantly different. Vertebral FF was moderately negatively correlated with aBMD, especially in women (*r* = -0.638, *p* < 0.001). R2* was mildly to moderately positively correlated with aBMD in men (*r* = 0.350, *p* = 0.003), but not in women. Moreover, FF was positively correlated with age, R2* was negatively correlated with age in men, and BMD was negatively correlated with age.

**Conclusions:**

The vertebral FF value of IDEAL-IQ sequence has the potential to be a new biological marker for the assessment of osteoporosis. Vertebral FF is moderately negatively correlated with aBMD, especially in women, allowing accuratly quantify the bone marrow fat. R2* value is mildly to moderately correlated with BMD in men and can be served as a complementary tool in the assessment of osteoporosis.

## Introduction

Osteoporosis(OP) is defined as a systemic metabolic bone disease that is characterized by decreased bone mass and microarchitectural deterioration of bone tissue, which leads to increased bone fragility and elevated fracture risk. As of August 2020, the global prevalence of osteoporosis was approximately 18.3%, with the prevalence in women at approximately 23.1% and in men at about 11.7% [1]. In 2005, more than 2 million fragility fractures occurred in the United States, costing $17 billion, and the incidence of fractures and the expenses are predicted to increase by nearly 50% by 2025 [2]. Osteoporosis has become a global public health problem.

The diagnosis of osteoporosis is based on bone mineral density (BMD), which is mainly measured by dual energy X-ray absorptiometry (DXA) and quantitative computed tomography (QCT) [3]. DXA, as a traditional and practical method, has some advantages of low cost, less radiation, convenience and high repeatability. However, the areal bone mineral density (aBMD) obtained through the method is susceptible to a variety of factors, including spinal degenerative changes, scoliosis, aortic calcification, and other abdominal calcifications [4]. QCT measures volumetric bone mineral density (vBMD), which can get rid of the influence of above factors and is more sensitive and accurate in diagnosing osteoporosis [5]. Although BMD has been considered the gold standard for assessing bone strength and predicting fragility fractures, bone strength is also related to composition, microstructure, mineralisation and microcirculation of the bone and so on [6].

Bone marrow adipose tissue (BMAT), one of the major components of bone, is increasingly known to all because of the important role in the development of osteoporosis. There is a competitive inhibitory relationship between bone marrow adipocytes and osteoblasts [7]. It is of interest for many researchers to utilize bone marrow fat to diagnose osteoporosis and assess the fracture risk. Non-invasive MRI quantification techniques of BMAT mainly include magnetic resonance spectroscopy (MRS) and chemical shift-encoded MRI (CSE-MRI) hydrolipid separation techniques, the latter mainly performed with Philips’ mDIXON-Quant sequence, Siemens’ Dixon -VIBE sequence, GE’s IDEAL-IQ sequence and so on. MRS is considered by many scholars to be the gold standard for the quantitative assessment of fat composition in vivo; however, it is extremely limited in skeletal system disease due to its long scan time and complex post-processing [8]. With the advent of CSE-MRI, rapid and accurate assessment of bone marrow fat has become possible, and the results match well with MRS measurements [9, 10]. This indicates that water–lipid separation technology has unquestionable feasibility and application value in clinical practice.

The iterative decomposition of water and fat with echo asymmetrical and least-squares estimation quantitation sequence (IDEAL-IQ sequence) is a new sequence developed from Dixon techniques, which utilizes a six-echo FSE sequence. The water image, fat image, in-phase and out-phase image, fat fraction map and R2* map can be obtained in a single scan, and the sequence has removed influence of T2* effect, T1 relaxation effect, eddy current effect and the confounding factors of fat multispectral peak models [11, 12]. The fat fraction (FF) value measured on the fat fraction map is utilized to quantify vertebral bone marrow fat and R2* value obtained from the R2* map to reflect iron deposition in vivo, and some studies have pointed out that bone marrow fat component and iron deposition have effects on the process of bone formation and resorption.

This study aimed to determine the relationship between FF, R2* value of IDEAL-IQ sequence and BMD value, and to explore their application value in the osteoporosis, with DXA as reference standard.

## Materials and methods

### Study population

This prospective study conformed to the Declaration of Helsinki, and was approved by the Ethics Committee of the First Affiliated Hospital of Chongqing Medical University(2019–140). All subjects signed written informed consent forms, and completed DXA and MRI examination of lumbar at the same day. A total of 174 subjects with a mean age of 53.63 ± 12.10 years were enrolled from September 2022 to May 2023. There were 105 females (mean age 53.47 ± 11.96 years) and 69 males (mean age 54.46 ± 12.55 years). The subjects were divided into normal, osteopenia, and osteoporosis group, according to the aBMD determined by DXA examination.

The inclusion criteria included females and males aged over 30 years who voluntarily underwent DXA and MRI examination. The exclusion criteria were as follows: people with lumbar spine tuberculosis, vertebral tumors, or surgery; people with other bone metabolic or endocrine diseases, such as hyperparathyroidism; people currently taking medications that may affect bone metabolism, such as glucocorticoids and bisphosphonates; and people with metal implants in their body or claustrophobia, which would prevent them from participating in MRI examination.

### Methods

#### DXA scan

A DXA scanner (Discovery A, Hologic Inc., USA) was used to measure the aBMD value and T-score of the L1–L4 vertebrae, with the scanning parameters voltage 140/100 kV and current 2.5 mA. According to the diagnostic standards recommended of T-score by the World Health Organization (WHO): normal bone mass [T-score ≥  − 1.0 standard deviation (SD)], osteopenia (− 2.5 SD < T-score <  − 1.0 SD), and osteoporosis (T-score ≤  − 2.5 SD) [13].

#### MRI examination

**Fig. 1:**
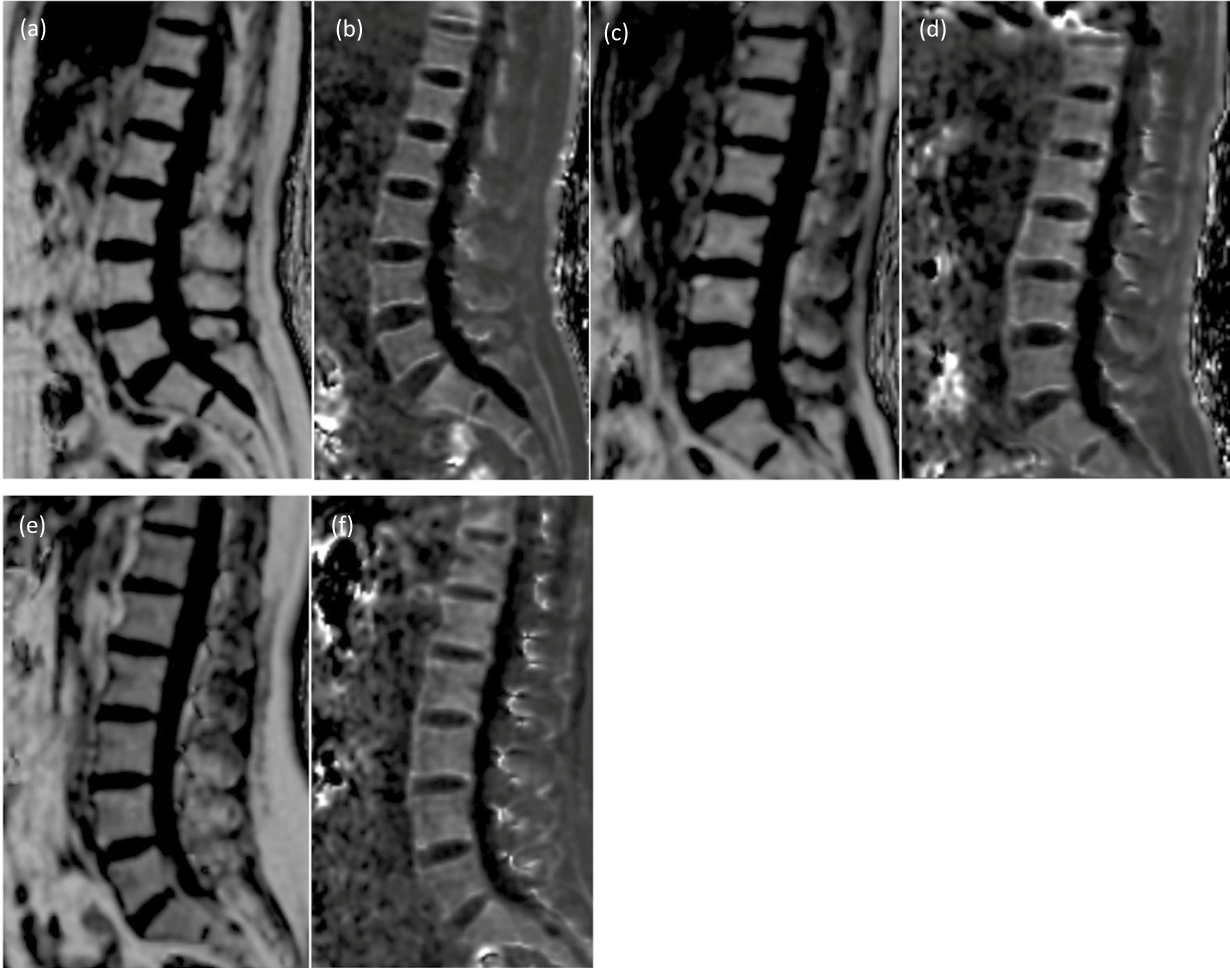
The following images are FF maps and R2* maps of three females. **a**, **b** is a 66-year-old woman with mean FF and R2* value of 71.66 and 151.54 Hz, and is diagnosed as osteoporosis. **c**, **d** is a 57-year-old woman with mean FF and R2* value of 58.66% and 170.36 Hz, diagnosed as osteopenia. **e**, **f** is a 49-year-old woman with mean FF and R2* value of 50.28% and 162.97Hz, diagnosed as normal bone mass

A 3.0 T MR scanner (Discovery 750, GE, USA) was used. The scanning parameters for the IDEAL-IQ sequence were as follows: TR 6.3 ms, TE 2.9 ms, FOV 38 cm × 38 cm, slice thickness 5 mm, matrix 16 cm × 16 cm, flip angle 4°, NEX 2, number of slices 72, and scan time of the sequence 14 s. After the MRI examination was completed, the L1–L3 vertebral FF and R2* value were measured independently by two radiologists with 2 years of experience using a GE AW 4.6 post-processing workstation. On the FF and R2* map, the sagittal midplane was selected, and the rectangular ROI (trying to include the entire cancellous bone of the vertebral body while avoiding the cortical bone, intervertebral discs, and vertebral veins) was manually outlined to directly obtain the FF and R2* value of each vertebra, and calculated the average FF and R2*. FF maps and R2* maps of three females are shown in Fig. 1.

In addition, 30 females and 30 males were randomly selected from all data sets using a random table generated by SPSS to measure FF and R2* value and assess intra-observer reliability. These 60 subjects were also analyzed by another radiologist with 2 years of experience to investigate inter-observer reliability.

### Statistical analysis

Statistical software (IBM SPSS Statistics, version 26.0, Armonk, NY, USA) was used for data analysis. The data conforming to normal distribution were expressed as mean ± standard deviation (SD). The differences among normal, osteopenia and osteoporosis group in terms of normal variables were compared through One-way ANOVA. Independent samples t-test was utilized to compare FF and R2* value between men and women. Pearson correlation test was applied for analyzing the correlation between two different parametric variables. Reproducibility of the data was assessed by the root mean square coefficient of variation (RMS-CV), and inter- and intra-observer agreement of measurements between two radiologists was assessed by intraclass correlation coefficient (ICC).Differences were considered statistically significant at a *P*-value less than 0.05.

## Results

### Reproducibility test of FF and R2* value

The reproducibility of the FF and R2* measurements was satisfactory, with RMS-CV of 2.41% and 2.63%, respectively. The inter- and intra-observer agreements of FF and R2* value were excellent, with ICCs ranging from 0.971 to 0.990 (> 0.9).

### Comparison of clinical and radiologic characteristics of all subjects among different bone mass groups

With DXA as diagnostic criteria, there were significant differences in age, height, weight, T-score, aBMD and FF value among the normal, osteopenia and osteoporosis group, except for BMI and R2* value (Table 1).

**Table 1:**
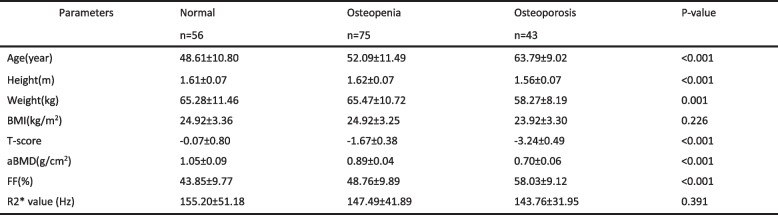
Clinical and radiologic characteristics of all subject

Data are expressed as mean ± standard deviation

*BMI* Body mass index, *aBMD* Areal bone mineral density, *FF* Fat fraction

### Comparison of FF, R2* value between groups of different gender, age and menopausal status

No significant difference was found in FF value between male and female group, while R2* value were significantly different (Table 2). Men were divided into age < 50 years and age ≥ 50 years group according to age, and women were divided into non-menopausal and post-menopausal group according to whether they were menopausal. And there were significant differences in both FF and R2* value between different groups (*P* < 0.05) (Table 3).

**Table 2:**
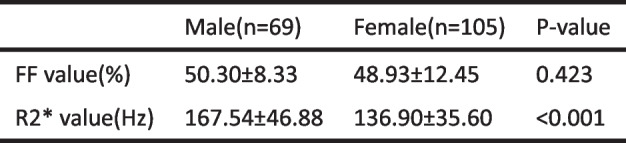
Comparison of FF, R2* value between males and females

**Table 3:**
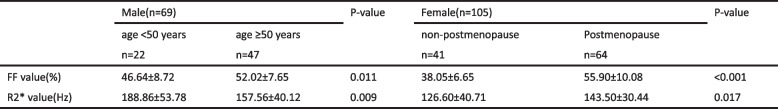
Comparison of FF, R2* value between groups of different gender, age and menopausal status

### Correlation between age and T-score, aBMD, FF and R2* value

Age was negatively correlated with T-score and aBMD value, and the correlation coefficients were -0.498 and -0.494, *p* < 0.001. FF was positively correlated with age (*r* = 0.643, *P* < 0.001). However, R2* value exhibited no significant correlation with age. The results are shown in Table 4, and indicate that there is a decreasing trend in BMD with aging while an ascending trend in FF.

**Table 4:**
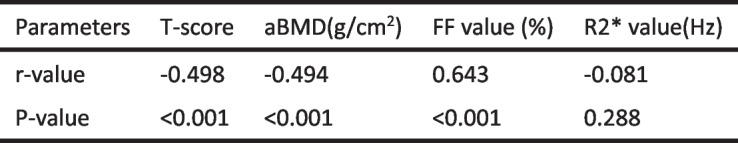
Correlation between age and T-score, aBMD, FF and R2* value

### Correlation between FF, R2* value and T-score, aBMD value

Vertebral FF was negatively correlated with T-score and aBMD, with correlation coefficients of approximately: r_1_ = -0.527, P_1_ < 0.001; r_2_ = -0.515, P_2_ < 0.001 respectively (Table 5). R2* was weakly correlated with aBMD (*r* = 0.192, *P* = 0.011), but there was no correlation with T-score.

**Table 5:**
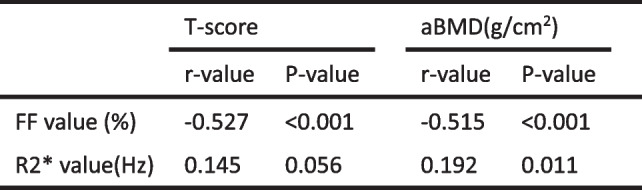
Correlation between FF, R2* value and T-score, aBMD value

### Correlation between FF, R2* value and age, T-score, aBMD, FF or R2* value in different groups by gender

**Fig. 2:**
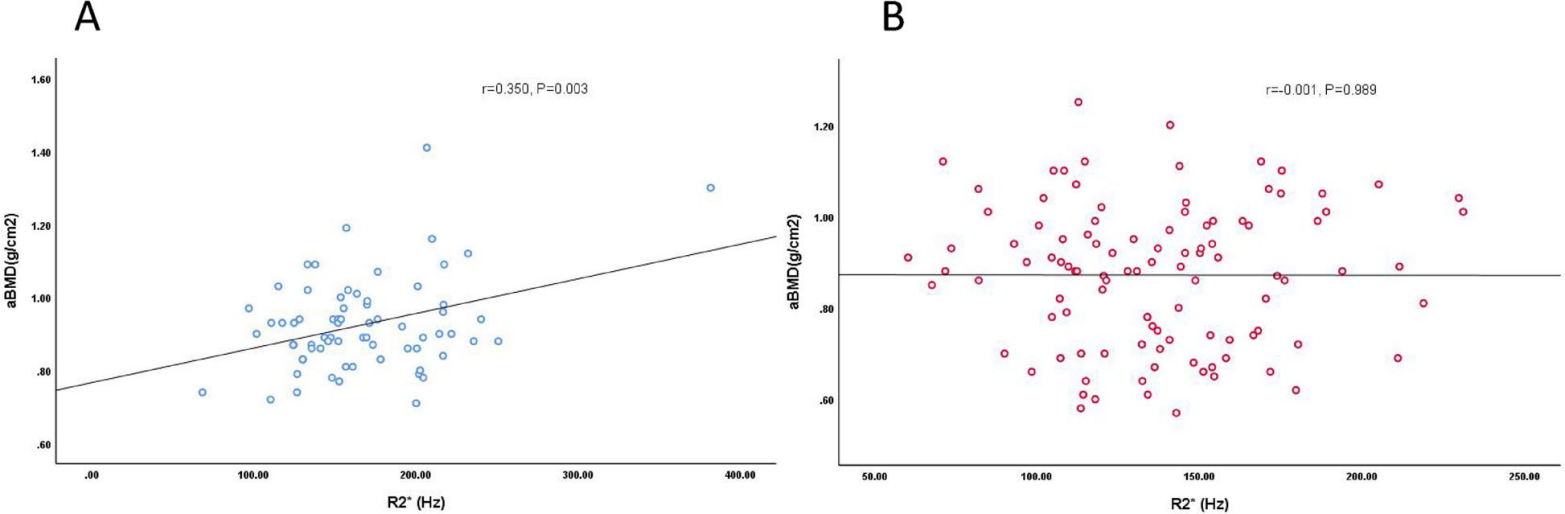
R2* value of male (**A**) is mildly to moderately positively with aBMD value, while R2* value of female (**B**) has no relation with aBMD value

**Fig. 3:**
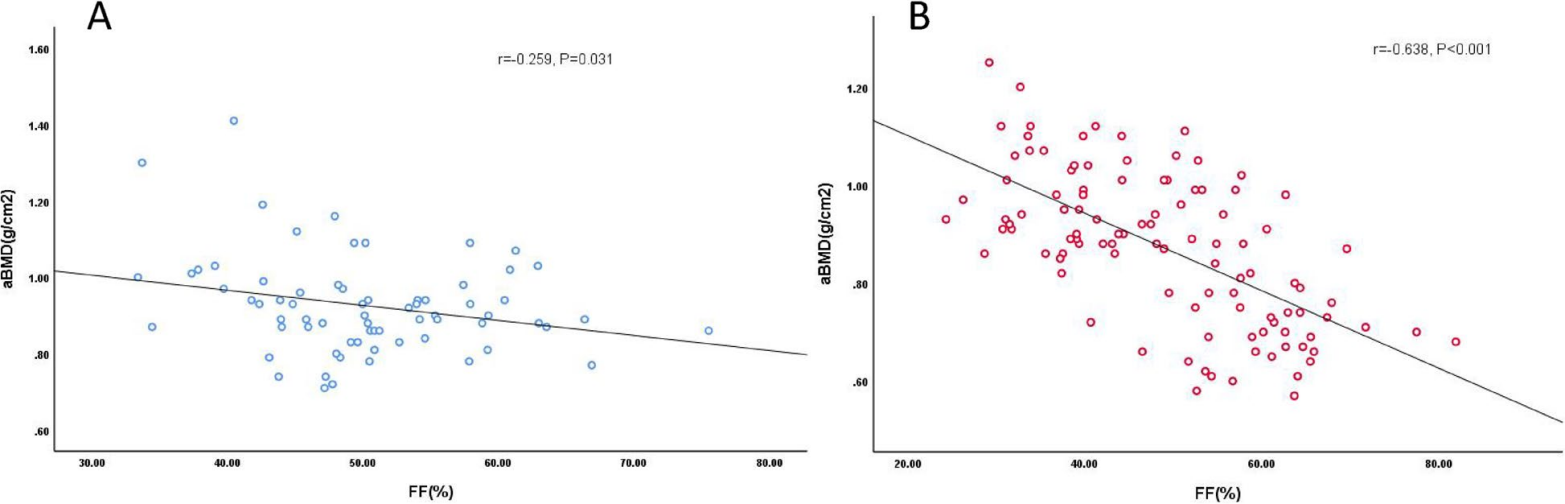
FF value of male (**A**) is weakly negatively correlated with aBMD value, and FF value of female (**B**) is moderately negatively correlated with aBMD value

In the male group, R2* was negatively correlated with age (*r* = -0.409, *P* < 0.001), positively correlated with T-score and aBMD value, but no correlation with FF (Table 6, Fig. 2). The correlations between R2* value and other parameters were extremely weak in female group. In addition, the correlation coefficients between FF value and age, aBMD in females were 0.725 and -0.638 respectively, while in males the correlation coefficients were 0.506 and -0.259, which meaned the correlations were more significant in women than in men, and FF was weakly positively correlated with R2* in women (*r* = 0.260, *p* = 0.007) (Table 6, Fig. 3).

**Table 6:**
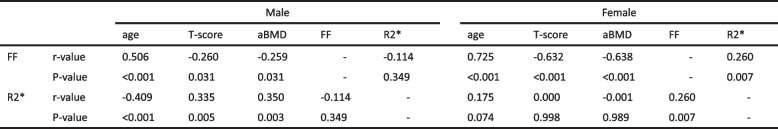
Correlation between FF, R2* value and age, T-score, aBMD, vBMD, FF or R2* value in different groups by gender

## Discussion

IDEAL-IQ sequence is a new MRI quantitative technique with the parameters FF value, which accurately quantifies bone marrow fat, and R2* value, which reflects iron deposition in vivo*,* contributing to indirectly estimate bone density and strength [14]. Vertebral FF value can be capable of playing an important role in the diagnosis of osteoporosis, prediction of fragility fractures and differentiating osteoporotic fractures from malignant fractures [15, 16]. Therefore, IDEAL-IQ sequence has the potential to be an additional tool for diagnosing osteoporosis.

Bone mineral density explains approximately 70% of bone strength, while the remaining 30% is affected by other elements, of which bone marrow adiposity is closely related to bone density and bone strength. Bone marrow adipocytes and osteoblasts are both derived from mesenchymal stem cells (MSCs), and there is a competitive inhibitory relationship between them. With aging, oestrogen deficiency and glucocorticoid exposure, MSCs preferentially differentiate into adipocytes, resulting in reduced bone formation [17]. Some studies [18] have verified that reduced bone mineral density was closely associated with increased bone marrow adiposity.

Iron deposition has also been indicated to depress bone density and strength, and raise the incidence of osteoporosis [19]. Iron metabolism plays an important role in bone homeostasis. Not only does disorder of iron metabolism promote osteoclast differentiation and osteoblast apoptosis, but also inhibit the proliferation and differentiation of osteoblast, ultimately leading to an imbalance between bone formation and resorption [20, 21]. The T2* MRI technology has been considered to be one of the main imaging examinations to evaluate iron deposition, and R2* value of IDEAL-IQ sequence equals to 1/T2* [22]. The correlation between R2* value and liver iron concentration has been more extensively studied, and T2* MRI technology has the advantages of short acquisition time, and the ability to quantify liver steatosis and iron overload simultaneously [23]. However, R2* is rarely applied in osteoporosis currently. We would investigate the correlation between FF, R2* value of IDEAL-IQ sequence and BMD and explore their application value in the osteoporosis.

Significant differences were found in age, height and weight among different bone mass groups. Bone loss was more pronounced in subjects who tended to be short and thin, and osteoporosis was more likely to occur. Fahimfar et al. [24] found that BMI was negatively associated with osteoporosis in both men and women aged over 60 years. In addition, vertebral FF value was significantly larger in the OP group than in the normal and osteopenia group [25–27].

No significant difference was found in FF value between male and female, while R2* value was obviously different. There was variability in both FF and R2* value between men aged < 50 years and ≥ 50 years and between menopausal and non-menopausal women, suggesting that FF was primarily age-related, while the difference in R2* may be due to difference in gender. When subjects were not divided by gender, age was negatively correlated with T-score and aBMD value (*r* = -0.494 ~ -0.498, *P* < 0.001), positively correlated with FF value (*r* = 0.643, *P* < 0.001) but not correlated with R2* value. When subjects were divided by gender, R2* value of males was negatively correlated with age, and mildly to moderately positively correlated with aBMD, while R2* value of females was not significantly correlated with other parameters. The correlation coefficients between FF value and age, aBMD in females were 0.725 and -0.638 respectively, while in males the correlation coefficients were 0.506 and -0.259, which meaned the correlations were more significant in women than in men.

At present, the correlation analyses between FF and BMD value are relatively common. Zhao et al. [28] found there were significant difference in FF among different bone mass groups, with FF moderately inversely correlated with vBMD after controlling for age, gender and BMI. Liu et al. [29] found a strong negative correlation between FF and aBMD value (*r* = -0.93,*p* < 0.001), which was much higher than our results (*r* = -0.515,*P* < 0.001). We speculated that the age of included participants over 50 years and the small sample size may be responsible for this. Chang et al. [30], adopting MRS and mDixon- Quant to measure FF value, found that both of two were inversely correlated with BMD (Y = -0.1906*X + 75.08/ Y = -0.1201*X + 69.15). In addition, FF was positively correlated with age, while BMD exhibited a negative correlation with age. The results of our study are roughly similar to the above studies, though the correlation coefficients may be different due to the difference in included population and the inconsistence in diagnostic criteria selected.

In recent years, quantitative assessment of FF and R2* value of lumbar spine has been performed, and FF and R2* are considered to be probably moderate markers of osteoporosis [31, 32]. R2* map is obtained with T2* correction, and R2* value is equivalent to 1/T2*. In patients with osteoporosis, the decay of T2* is delayed due to loss of bone trabeculae, which leads to a decrease in R2* [33]. Previous studies [34, 35] have found significant differences in FF and T2* value among different BMD groups of QCT, with FF and T2* both negatively correlated with vBMD and both being effective in differentiating normal from abnormal BMD. Li et al. [36] found a moderately positive correlation between R2* and BMD in postmenopausal women, with the area under the curve (AUC) of 0.821, 0.784 and 0.922 for FF, R2* and FF combined with R2* for the diagnosis of osteoporosis. Liu et al. [37] found that R2 * value was weakly associated with FF and BMD (*r* = -0.219, 0.290, *P* < 0.05), and the diagnostic efficacy of R2 * in diagnosing osteoporosis was relatively poor. Kim et al. [38] selected L4 vertebra to measure FF and R2* value and found that R2* correlated more significantly with BMD in women than in men, with higher AUCs for FF in men and R2* in women when predicting osteopenia and osteoporosis.The different results of different studies may be due to a variety of reasons including variability in the included participants, inconsistency in the range of vertebrae studied, and differences in the scanning protocols.

There are several limitations in our study. Firstly, the sample size was relatively small and the number of patients with osteoporosis was relatively insufficient, especially in men. Secondly, MRI examination included L1-L3 vertebrae while DXA measured aBMD of L1-L4 vertebrae, and the difference may influence the findings. What’s more, the incidence of vertebral fractures in our study was particularly low and did not allow for assessment of fragility fracture risk, which has been a hot topic in the research of osteoporosis currently.

## Conclusion

In short, IDEAL-IQ sequence is a fast, convenient, non-invasive and non-radiation technique. The parameter FF performs a more significant correlation with T-score and aBMD in women than in men, therefore, FF value has the potential to be a new biological marker for the assessment of osteoporosis, predicting the risk of fragility fractures and assessing the efficacy of treatment in women. In contrast, R2* value is mildly to moderately correlated with BMD in men and can be served as a complementary parameter to FF and BMD in the assessment of osteoporosis.

## Data Availability

The datasets generated and/or analyzed during this study are not publicly available due to personal protection laws. Subsets or aggregation of these data will not include information that could compromise the research participants’ privacy and are available from the corresponding author upon reasonable request.

## Abbreviations

BMD: Bone mineral density
aBMD: Areal bone mineral density
vBMD: Volumetric bone mineral density
DXA: Dual energy X-ray absorptiometry
QCT: Quantitative computed tomography
IDEAL-IQ: Iterative decomposition of water and fat with echo asymmetry and least-squares estimation quantitation sequence
FF: Fat fraction
